# Research on the construction of corneal endothelium transplantation with acellular amniotic membrane as a scaffold

**DOI:** 10.3389/fmed.2025.1592123

**Published:** 2025-07-01

**Authors:** Ya-Nan Chen, Rui-Qin Guo, Bo-Yu Liang, Hong-Qin Ke, Meng-Jie Dong, Ming-Fang He, Ji Yang, Hai Liu

**Affiliations:** Department of Ophthalmology, The Eye Disease Clinical Medical Research Center of Yunnan Province, The Eye Disease Clinical Medical Center of Yunnan Province, Second People’s Hospital of Yunnan Province, The Affiliated Hospital of Yunnan University, Kunming, China

**Keywords:** human acellular amniotic membrane, corneal endothelium, decellularized, bioanalysis, cytocompatibility

## Abstract

**Introduction:**

This study aimed to develop a human acellular amniotic membrane (HAAM) scaffold suitable for corneal endothelial transplantation. The HAAM was engineered using sequential chemical treatments and physical agitation to remove cellular components while preserving the extracellular matrix structure. The study sought to evaluate the biocompatibility and functional properties of the HAAM when seeded with immortalized human corneal endothelial cells (HCECs), with the ultimate goal of providing a potential therapeutic option for corneal endothelial dysfunction.

**Methods:**

The HAAM was fabricated through a series of chemical treatments involving trypsin/EDTA, Triton X-100, sodium deoxycholate, and peracetic acid/ethanol, combined with physical agitation. Following lyophilization, the HAAM was sterilized and coated with fibronectin and chondroitin sulfate (FNC) to enhance cell adhesion. HCECs were then seeded onto the HAAM scaffold. Biocompatibility was assessed by evaluating cell adhesion using microscopy, cell viability using CCK-8 and EdU assays, and cell proliferation. Functional validation included immunofluorescence detection of tight junction proteins (ZO-1), transcriptome sequencing (RNA-seq), and quantitative PCR (qPCR) to analyze the expression of genes regulating barrier function, ion transport, and extracellular matrix synthesis. Additionally, the expression of key genes critical for endothelial function was assessed to validate the functionality of the HAAM-based corneal endothelial transplantation membrane.

**Results:**

The HAAM was successfully prepared, maintaining an intact collagen fiber structure. HCECs adhered closely to the HAAM scaffold, forming a continuous monolayer. The HAAM promoted cell viability and proliferation, as evidenced by positive expression of tight junction proteins and upregulation of key functional genes. Transcriptome analysis identified genes involved in proliferation and matrix synthesis, further supporting the biocompatibility and functional properties of the HAAM.

**Discussion:**

The HAAM scaffold demonstrated excellent transparency, mechanical properties, and biocompatibility, making it suitable for the attachment and proliferation of HCECs. The effective maintenance of key functional gene expression levels suggests that the HAAM functionally mimics the characteristics of the natural corneal endothelial layer. These findings provide experimental evidence for the potential clinical application of the HAAM in corneal endothelial transplantation, offering a promising therapeutic option for patients with corneal endothelial dysfunction. Further studies are warranted to explore the long-term efficacy and safety of the HAAM in preclinical and clinical settings.

## 1 Introduction

The corneal endothelium plays a crucial role in maintaining the normal moisture, thickness, and transparency of the cornea (1), while the human corneal endothelium has almost no regenerative capacity *in vivo* (2). Therefore, severe damage to the corneal endothelial cells will lead to irreversible corneal edema. Based on the data from the World Health Organization (WHO), corneal diseases represent one of the leading causes of blindness globally. Impairment of endothelial cell function can result in irreversible vision loss (3). Currently, allogeneic corneal transplantation using tissues derived from human donors remains the best option for treating blindness caused by corneal diseases (4). Traditional approaches such as penetrating keratoplasty (5) and endothelial keratoplasty (4, 6–8) are effective methods for treating blinding corneal diseases such as corneal endothelial decompensation. However, the current supply of corneal donors is far from meeting clinical demand (9–11). Therefore, identifying suitable seed cells to replace corneal endothelial cells and constructing corneal endothelial substitutes for transplantation therapy are critical solutions to address the clinical shortage of corneal endothelial donors (12, 13).

Isolating and expanding corneal endothelial cells *in vitro* is highly challenging, primarily due to the difficulty in proliferating them to sufficient numbers without undergoing premature senescence or endothelial-to-mesenchymal transition (14). Currently, researchers are also exploring alternative cell sources for corneal endothelial transplantation, such as vascular endothelial cells (15), bone marrow-derived mesenchymal stem cells (16), and skin-derived precursor cells (17). For the transplantation of corneal endothelial replacement cells, intracameral suspension cell injection and precipitation methods are currently the most commonly used approach (18, 19). However, suspended cells lack polarity, and their movement within the anterior chamber is uncontrolled, making it impossible for this transplantation method to ensure that the transplanted cells adequately adhere and grow on the corneal posterior elastic layer or posterior stromal layer. Additionally, the intracameral cell injection method may obstruct the trabecular meshwork, leading to increased intraocular pressure in the transplanted eye (20–23). Therefore, the development of a biocompatible scaffold that supports the adhesion and growth of transplanted cells and the co-culture of endothelial cell substitutes with the scaffold material prior to transplantation are prerequisites for the successful transplantation of corneal endothelial replacement cells.

The amniotic membrane is a transparent and resilient tissue that is devoid of nerves, blood vessels, and lymphatic vessels. Due to its properties of immune privilege, antimicrobial activity, and pain relief, allograft amniotic membrane transplantation has been utilized in surgical procedures to replace diseased or damaged tissues. It is also used to cover chronic wounds on the skin, cornea, and conjunctiva to promote healing (24–26). Furthermore, the immune privilege of the amniotic membrane is tissue-specific and is often utilized in ophthalmology, particularly for the cornea, which also possesses immune privilege. It promotes epithelial cell growth (27), reduces inflammation (28), and inhibits neovascularization (29), making it a common biomaterial for ocular surface reconstruction. Human acellular amniotic membrane (HAAM) is a natural extracellular matrix material obtained by removing both amniotic epithelial cells (AECs) and amniotic mesenchymal stromal cells (AMSCs), as supported by Khosravimelal et al. (30) from fresh human amniotic membrane. During this process, not only are the epithelial cells removed, but also immunogenic cellular components, such as epithelial cells and potentially other cell types (e.g., AMSCs), thereby effectively reducing their potential immunogenicity while preserving the key components of the basal layer and extracellular matrix. Moreover, the exposure of the basement membrane facilitates cell adhesion and proliferation (31). Currently, HAAM has been widely used in various medical fields, such as skin defect repair (32), peripheral nerve regeneration (33), and cartilage injury treatment (34), achieving remarkable therapeutic effects. With the continuous innovation of research techniques and the accumulation of extensive research data, acellular amniotic membrane is increasingly being applied in the field of ophthalmology (35, 36), although it is primarily focused on the conjunctiva and corneal epithelium, for example, in conjunctival sac plasty (37) and in supporting the growth of human limbal epithelial cells, immortalized corneal epithelial cells, and induced pluripotent stem cells (38, 39). Its application in the corneal endothelium is still relatively rare.

In this study, we co-cultured immortalized human corneal endothelial cells (HCEC-B4G12) with the HAAM to construct a novel corneal endothelial graft. The HAAM scaffold was prepared through the SDS-free decellularization protocol, combining sequential chemical treatments with physical agitation, effectively preserving the collagen fiber structure while eliminating immunogenic components. This study pioneers the application of HAAM as a scaffold for corneal endothelial regeneration, offering a promising solution to both the shortage of donor tissue and the limitations associated with traditional cell injection methods.

## 2 Materials and methods

### 2.1 Materials

The immortalized human corneal endothelial cell line HCEC-B4G12 was purchased from Guangzhou Cellcook Biotech Co., Ltd. The acellular amniotic membrane was prepared in our laboratory using a fresh amniotic membrane. The human endothelial-SFM medium and trypsin were purchased from Gibco, United States. The coating solution and digestion termination solution specifically for HCEC-B4G12 were obtained from Guangzhou Cellcook Biotech Co., Ltd. The placental tissues were obtained from five healthy donors (approved by Yunnan University Ethics Committee, No. 2020087-88).

### 2.2 To establish the culture system of HCEC-B4G12

To establish the culture, immortalized human corneal endothelial cells, HCEC-B4G12, were purchased cells from Cellcook Company. The culture of corneal endothelial cells *in vitro* often requires the addition of extracellular matrix components and growth factors to the culture medium, such as chondroitin sulfate, carboxymethyl chitosan, EGF, bFGF, and NGF. The addition of extracellular matrix components and growth factors plays an important role in promoting the adhesion of corneal endothelial cells (40–42). In this study, a mixture of 10 μg/mL of fibronectin and 10 mg/mL of chondroitin sulfate (FNC) was used to pre-treat the amniotic membrane material to facilitate cell attachment and growth. In addition, 2–3 mL of FNC was added to a T25 culture flask, and then, the flask was placed in a cell incubator at 37°C with 5% CO_2_ for 12 h. Before adding the cells, the FNC was aspirated, and the flask was washed once with PBS. Based on the growth habits and patterns of endothelial cells, a serum-free medium, human endothelial-SFM (containing 10 ng/mL of bFGF), was selected for culturing in a cell incubator at 37°C with 5% CO_2_. The cell culture medium was changed every other day.

### 2.3 Preparation and preservation of HAAM

**Figure 1:**
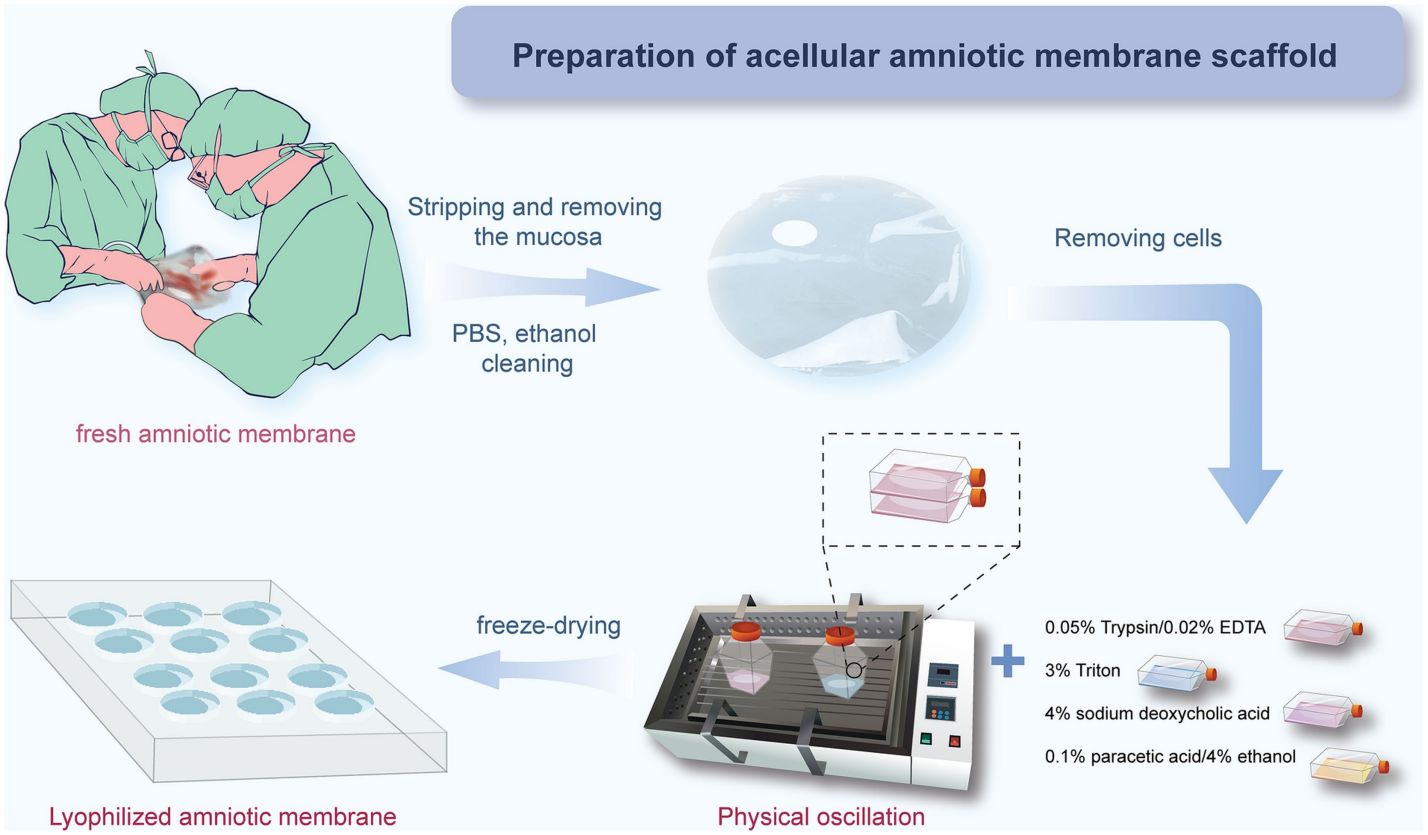
AM decellularization process flowchart.

**Figure 2:**
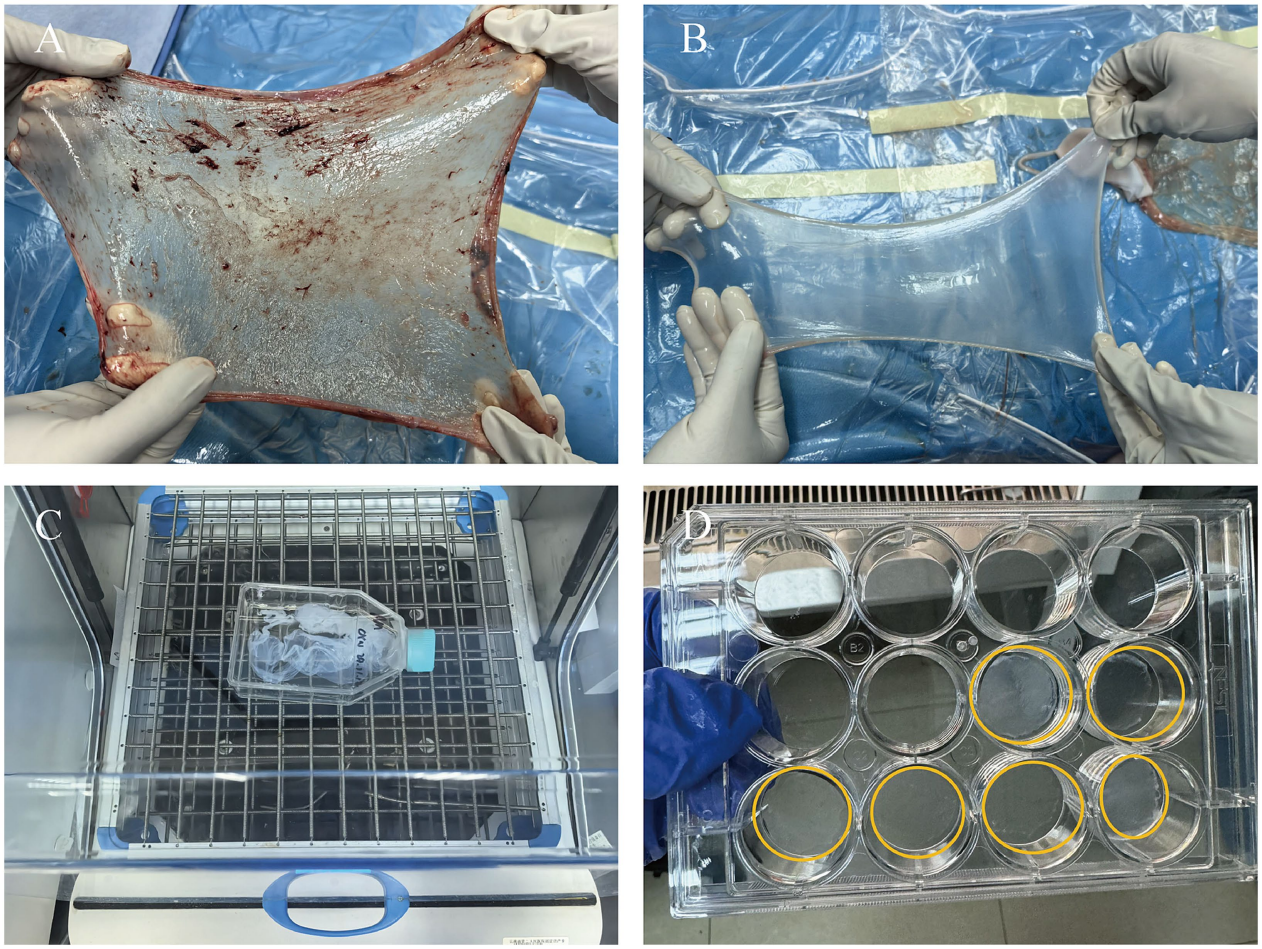
Process of preparing HAAM. **(A)** Fresh AM. **(B)** AM after separation and washing, presenting as a semi-transparent membrane. **(C)** AM undergoes oscillation treatment on a shaker. **(D)** AM after freeze-drying, with good transparency.

In this study, we used a combined approach of physical and chemical methods, aiming to completely remove the cellular components on the amniotic membrane surface, including amniotic epithelial cells (AECs) and amniotic mesenchymal cells (AMSCs), to avoid sensitization and immune responses, as well as to reduce the risk of infection (43), while maximizing the preservation of its natural extracellular matrix structure and biological activity (Figure 1). Approval was obtained from the Ethics Committee of Affiliated Hospital of Yunnan University, and informed consent forms were signed by the patients (Approval Number: 2020087 and 2,020,088). The specific steps are as follows: first, placenta was obtained from healthy pregnant women who had undergone cesarean section without any infectious diseases, and the amniotic membrane was removed. After sample collection, the fresh amniotic membrane was quickly placed in an ice box within 30 min for storage and transported back to the laboratory, where it was processed within 12 h to ensure the activity and integrity of the material. The membrane was rinsed with distilled water to remove blood and debris. Under strict sterile conditions, the amniotic membrane was separated from the chorion using forceps to remove the mucosal layer (Figures 2A,B). Subsequently, the membrane underwent multiple rounds of oscillation treatment using a constant temperature shaker (Bluepard, Shanghai) at 150 rpm and 37°C. It was then rinsed with sterile deionized water (Gibco, United States) on a shaker for three cycles, each lasting 10 min. Next, it was rinsed with 0.05% trypsin on the shaker for 1 h, followed by four cycles of rinsing with 3% Triton (Sigma-Aldrich, United States) on the shaker, each cycle lasting 1 h (Figure 2C). It was then rinsed with 4% sodium deoxycholate on the shaker for 1 h and subsequently rinsed with 4% ethanol (EMD Millipore, United States) for 4 h. Afterward, it was rinsed with PBS (Gibco, United States) for 15 min and then with sterile deionized water for two cycles, each lasting 15 min. After these steps, the amniotic membrane was thoroughly cleaned to remove all chemical reagent residues and then lyophilized for storage to maintain its structural stability. Before use, it was also subjected to epoxy treatment to eliminate potential microorganisms and ensure biological safety. To verify the effectiveness of the decellularization process, we used standard histological staining methods, namely hematoxylin and eosin (HE) staining and Masson’s trichrome staining.

### 2.4 To construct HCEC-HAAMs with acellular amniotic membrane as a scaffold

Acellular amniotic membrane scaffold was placed at the bottom of each well of a 12-well culture plate, and 1 mL of FNC was added to each well. The plate was then placed in a CO₂ incubator overnight. On the following day, the FNC was removed, and the wells were washed with PBS before adding a certain amount of culture medium. Immortalized human corneal endothelial cells were seeded onto the 12-well plate at a density of 1 × 10⁵ cells/mL. The medium was changed every other day. The morphology of the endothelial cells was regularly observed using an inverted phase-contrast microscope to assess whether the corneal endothelial cells could grow in a tightly packed arrangement on the scaffold material. After 4 days of culture, frozen sections were stained with HE to more intuitively demonstrate the integration of HCECs with the acellular amniotic membrane scaffold.

### 2.5 Observation under scanning electron microscope

To delve into the ultrastructural characteristics of HAAM, we used high-resolution scanning electron microscope (SEM) for detailed observation and analysis. Both the pure acellular amniotic membranes that had undergone freeze-drying and epoxy treatment, and biomimetic corneal endothelial grafts cultured for 4 days, were fixed with electron microscopy fixative in a dark environment at room temperature for 30 min each. After fixation, they were rinsed three times with 0.1 M phosphate buffer (PB) for 15 min each time. Subsequently, they were fixed with 1% osmium tetroxide prepared in 0.1 M PB in a dark environment at room temperature for 1 h, followed by three rinses with 0.1 M PB for 15 min each time. Finally, after dehydration and drying, they were coated with gold and observed using a scanning electron microscope.

### 2.6 Assessment of viability and proliferation capacity of corneal endothelial cells on HAAM

The CCK-8 assay was used to evaluate the viability of HCEC-B4G12 on acellular amniotic membrane scaffolds. The experimental design included multiple groups: HAAM + FNC + HCEC, HAAM + HCEC, empty well + FNC + HCEC, and empty well + HCEC. Cells from each group were seeded in 12-well culture plates at a density of 10^5^ cells/well with 2 mL of medium and incubated in a cell culture incubator at 37°C with 5% CO_2_ for 72 h. Then, 200 μL of CCK-8 solution (Proteintech, United States) was added to each well of the plate and incubated for an additional 2 h in the incubator. The absorbance was measured at 450 nm using a microplate reader (BioTeK, USA800TS). To further confirm the proliferative capacity of the endothelial cells, a 5-ethynyl-2′-deoxyuridine (EdU) assay was also performed. The EdU detection kit (Beyotime, Shanghai) was used according to the manufacturer’s protocol to assess cell proliferation. The cells were fixed with 4% paraformaldehyde (1 mL/well for 30 min), incubated with 10 μM EdU for 2 h, and permeabilized with 0.2% Triton X-100 (1 mL/well for 10 min). Subsequently, the cells were co-stained with DAPI and Apollo fluorescent dyes. Cell images were captured under a microscope.

### 2.7 Immunofluorescence detection of HCEC-HAAMs

To delve into the interactions between HCECs and HAAM in corneal endothelial grafts, particularly the expression of cell junction proteins, we used immunofluorescence staining techniques. HCEC-HAAMs cultured for 72 h were washed three times with PBS and fixed in 4% paraformaldehyde for 20 min. After aspirating the fixative, the cells were permeabilized with 0.3% Triton X-100 for 30 min. Then, 4% bovine serum (prepared in PBS) was added, and the plates were placed in a humidified chamber for 1 h. Primary antibody staining was performed by adding ZO-1 (1:100) to the wells. After washing the cells, they were incubated with FITC-labeled rabbit anti-goat IgG (1:100), RT-labeled rabbit anti-goat IgG (1:100), or goat anti-mouse IgG (1:10) secondary antibodies (prepared in PBS) for 2 h. Finally, the grafts were rinsed with PBS and observed and photographed under a fluorescence microscope.

### 2.8 RNA sequencing and analysis

Total RNA was extracted from cultured HCECs and identical cells grown on HAAM as described above. The integrity of the total RNA extracted from both samples was evaluated using an Agilent 2100 Bioanalyzer by Agilent Technologies. Subsequently, rRNA was eliminated from the total RNA to isolate the sample mRNA. This mRNA was then subjected to random fragmentation using divalent cations in the NEB fragmentation buffer, followed by chain-specific fragmentation for mRNA construction. Initial library quantification was carried out with a Qubit2.0 Fluorometer, and the library was subsequently diluted to a concentration of 1.5 ng/μL. The size of the library inserts was determined using an Agilent 2100 Bioanalyzer, and QRT-PCR was utilized to accurately quantify the library’s effective concentration, which needed to exceed 2 nM. After assessing the quality of the genomic DNA, it was fragmented through mechanical interruption (ultrasound). The fragmented DNA then underwent purification, end-repair, 3′ end adenylation, ligation to a sequencing adapter, and size selection using agarose gel electrophoresis. The resulting polymerase chain reaction (PCR) product was amplified to generate the sequencing library. Sequencing was conducted on the Illumina NovaSeq 6000 platform with a read length of 150 bp. Through quality control, trimming, deduplication, and alignment of the original Fastq data by a high-throughput sequencing service provider, we obtained the gene expression matrix of transcriptome sequencing. To detect DEGs, we used the “DESeq2” R package. The DESeq2 package was used for differential analysis of the original counts matrix, and the analysis was carried out according to the standard process. The variance stabilizing transformations (VST) method provided by the package DESeq2 was used to normalize the original counts matrix. A Wilcoxon rank-sum test was conducted to assess gene expression differences between the two sets of samples. Genes were considered significantly differentially expressed if they met the criteria of adjusted *p*-value of <0.05 and |logFC| >1. For data visualization, we utilized the “pheatmap,” “ggpubr,” and “ggplot2” R packages. To analyze the DEGs, we used the R tool known as “clusterProfiler” (44). Using the “clusterProfiler” R package, we carried out both Gene Ontology (GO) (45) and Kyoto Encyclopedia of Genes and Genomes (KEGG) analyses (46). Gene set enrichment analysis (GSEA) was conducted on all genes (previously ranked based on their log2FC between analyzed groups) using the clusterProfiler package. Enrichment was considered significant if the nominal false discovery rate (FDR) was <0.25 and the *p*-value was < 0.05, referencing the “c2.cp.all.v2022.1.Hs.symbols.gmt” gene set. These packages offer utilities for evaluating the enrichment or variability of gene sets within gene expression data, thereby enabling a thorough examination of pathway or gene set activities across different samples. *p*-values were calculated with the Benjamini–Hochberg method, and the terms with *p*-values <0.05 were considered to be significant.

### 2.9 Quantitative real-time PCR

HCECs cultured in dishes and HCECs grown on HAAM were lysed using a TRIzol reagent (Thermo Fisher, United States). Total RNA was extracted from these tissues using a Total RNA Extraction Kit (Vazyme, Nanjing, China). Subsequently, cDNA was synthesized through reverse transcription using a cDNA First-Strand Synthesis Kit (TaKaRa, Japan). The expression levels of genes were assessed using RT-qPCR with TB Green Fast qPCR Mix (TaKaRa, Japan). Gene expression was quantified relative to GAPDH using the 2^−ΔΔCt^ method. The primer sequences for these genes are provided in Supplementary Table 1.

### 2.10 Statistical analysis

The results are presented as mean ± SD. For two-sample comparison, a Student’s *t*-test was used. For multiple-sample comparison, analysis of variance (ANOVA) was performed to detect whether a significant difference existed between groups with different treatments using GraphPad Prism 9.5 (GraphPad Software, United States). A *p*-value of <0.05 was deemed statistically significant. In the graphs, asterisks are displayed to indicate the statistical significance of the values. ^*^p < 0.05, ^**^p < 0.01, and ^***^p < 0.001.

## 3 Results

### 3.1 Detection of the degree of amniotic membrane decellularization

**Figure 3:**
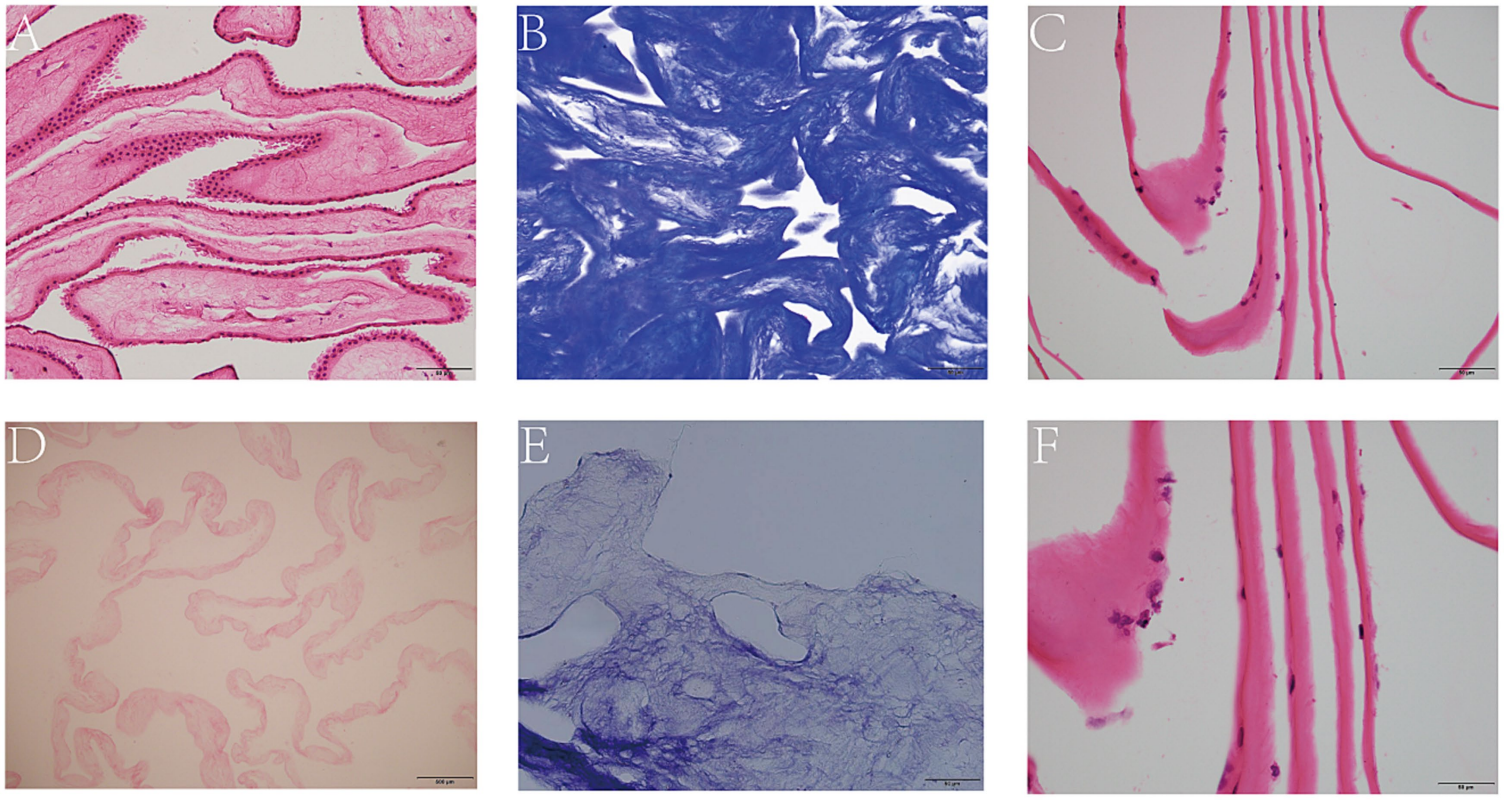
Decellularization of AM. HE staining of intact **(A)** and decellularized **(D)** AM. Masson staining of intact **(B)** and decellularized AM. **(E)** HE staining of HCEC-HAAMs **(C,F)**. Scale bar = 50 μm.

The lyophilized HAAM exhibited good light transmittance and adhered tightly to the bottom of the culture dish (Figure 2D). HE staining results visually demonstrated that the untreated amniotic membrane was covered with a dense layer of cells (Figure 3A), whereas the HAAM presented a smooth, cell-free surface (Figure 3D), confirming the effective removal of cellular components. Masson’s trichrome staining further revealed the fine structure of collagen fibers within the acellular amniotic membrane, demonstrating that the collagen fibers were tightly arranged in an orderly manner (Figures 3B,E).

### 3.2 Biocompatibility testing of HCEC-HAAMs

#### 3.2.1 Cell adhesion, morphology, and monolayer formation

**Figure 4:**
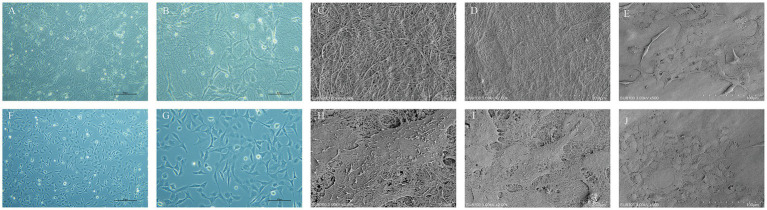
Growth condition of HCECs and the ultrastructure of HCEC-HAAMs. Cell growth on HAAM **(A,B)** and blank wells **(F,G)** 72 h after cell inoculation. Scale bar = 50 μm. **(C,D)** Scanning electron microscopy of the HAAM surface. **(C)** 5,000× magnification, scale bar = 10 μm. **(D)** 2,000× magnification, scale bar = 20 μm. **(E,H–J)** Scanning electron microscopy of cell-seeded membrane. The cells are visible as polygonal, predominantly hexagonal in shape, with microvilli on the surface, and the intercellular connections are relatively tight. 500× magnification. **(E,J)** Scale bar = 100 μm. **(D)** Scale bar = 10 μm. **(E)** Scale bar = 20 μm.

Using an inverted phase contrast microscope for dynamic observation, we captured real-time changes in the interaction between cells and the scaffold. The results demonstrated that HCECs began to attach to the HAAM 12 h after seeding, with cellular edges gradually extending pseudopodia to firmly adhere to the scaffold surface. As the culture time extended, the cell morphology underwent significant changes, transitioning from an initial round shape to a polygonal shape, demonstrating good spreading and viability. Further observation revealed that, by 48 h of culture, HCECs began to form distinct cellular colonies, which continued to expand and merge. At approximately 72 h, a continuous monolayer of cells was formed (Figures 4A,B,F–G). At this stage, the cells were regularly shaped and tightly arranged in an orderly manner. The results of frozen section HE staining showed that HCECs formed a monolayer on the scaffold, with clear cellular outlines, a moderate nucleus-to-cytoplasm ratio, and no significant cell necrosis or detachment observed (Figures 3C,F).

#### 3.2.2 Proliferation capacity of HCECs on HAAM

**Figure 5:**
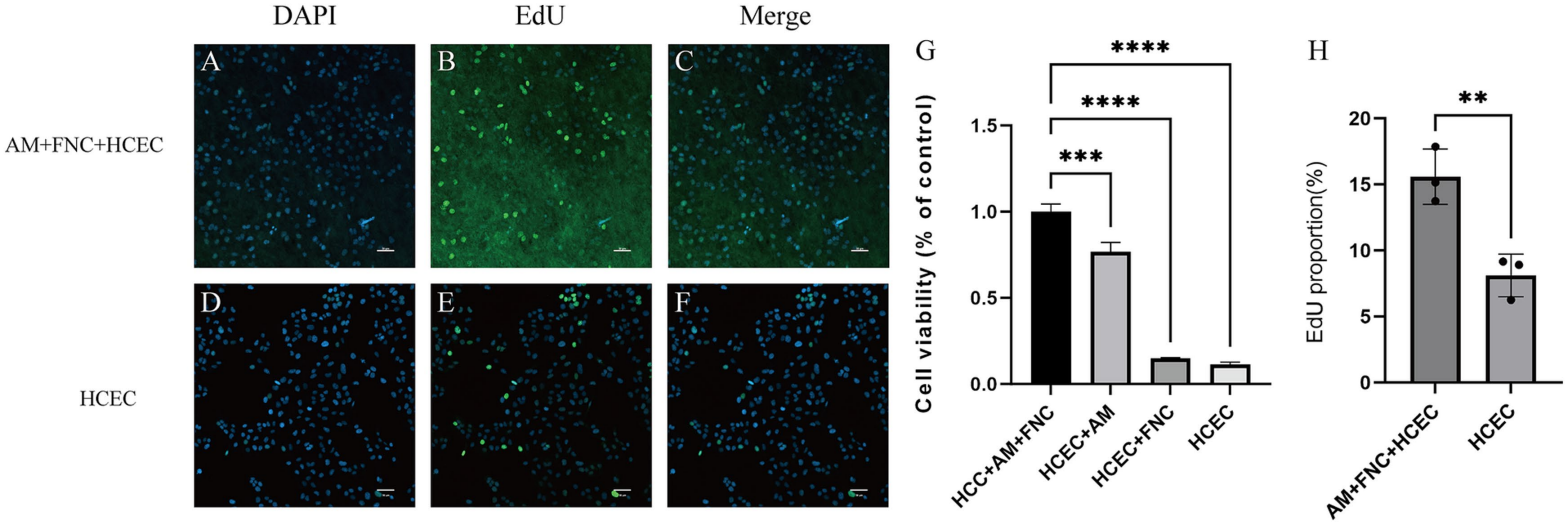
Proliferation ability of HCECs on HAAM. **(A–F,H)** EdU experiments also confirmed that HCECs grown on FNC-treated decellularized amniotic membrane scaffolds exhibited higher proliferative activity. **(G)** CCK8 assay showed that human corneal endothelial cells cultured on HAAM + FNC, HAAM alone, blank well + FNC, and blank well for 72 h, and the cells were most viable on HAAM + FNC.

The CCK8 results showed that, after culturing HCECs for 72 h on HAAM + FNC + HCEC, HAAM + HCEC, empty well + FNC + HCEC, and empty well + HCEC, the cells exhibited the highest viability on the amniotic membrane + FNC, indicating that culturing HCECs under these conditions can enhance their viability (Figure 5G). This finding not only verifies the promotive effect of the acellular amniotic membrane scaffold on HCEC growth but also reveals the synergistic effect of FNC as a growth factor in enhancing cell viability. The EdU assay confirmed that HCECs grown on the FNC-treated acellular amniotic membrane scaffold exhibited higher proliferative activity. Specifically, the EdU-positive rate (i.e., the proportion of proliferating cells) of these cells was significantly higher than that of cells grown on the bottom of a plain culture dish (Figures 5A–F,H).

#### 3.2.3 Immunofluorescence detection of HCEC-HAAMs

**Figure 6:**
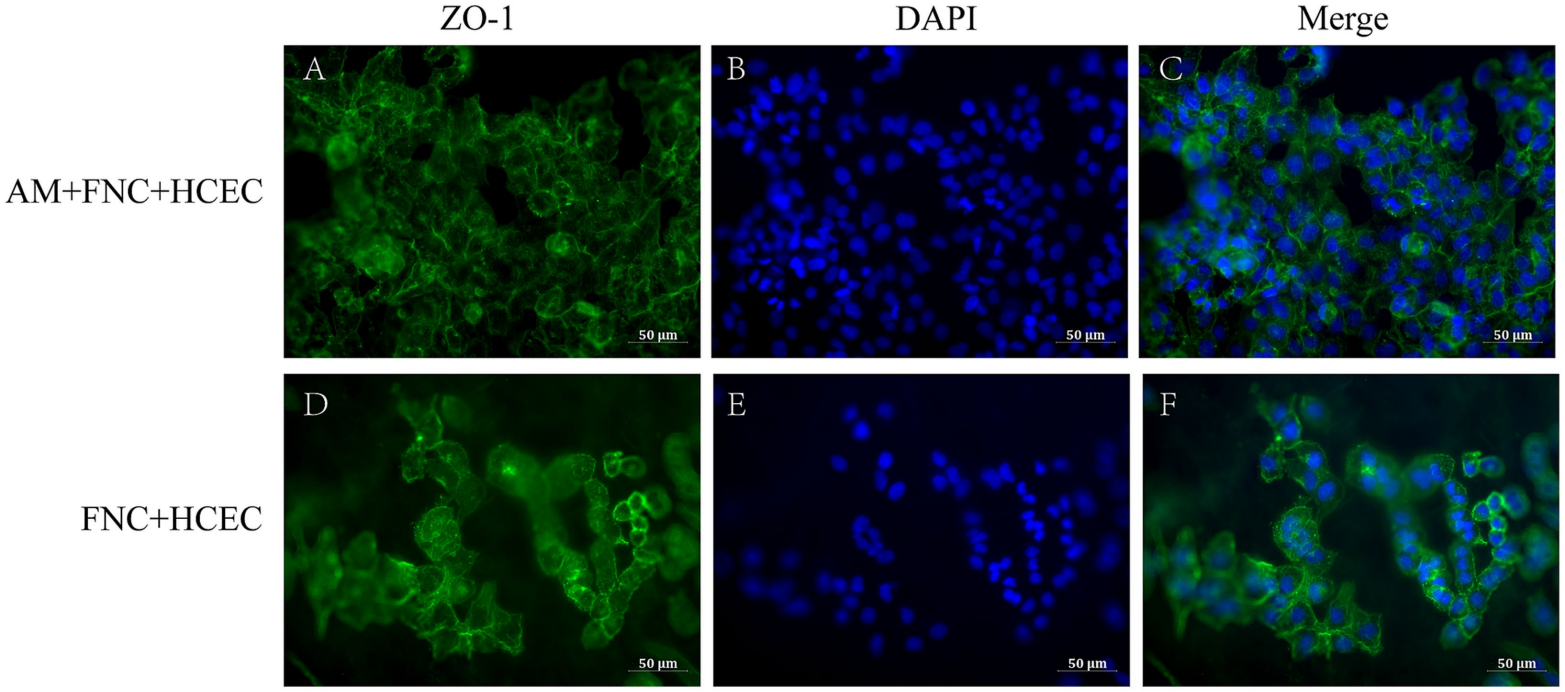
Immunofluorescence results. **(A–C)** The endothelial cells on the HAAM maintained positive expression of ZO-1**. (D–F)** The endothelial cells in the empty well + FNC + HCEC maintained positive expression of ZO-1.

The immunofluorescence results showed that, in the HAAM + FNC + HCEC group, ZO-1 exhibited robust and continuous linear staining along the cell boundaries, indicating the formation of stable tight junctions (Figures 6A–C). In contrast, in HCECs grown on regular culture dishes (FNC + HCEC), ZO-1 localization appeared fragmented (Figures 6D–F). As an integral component of tight junction proteins, the positive expression of ZO-1 confirms the formation of stable tight junctions between HCECs on the acellular amniotic membrane scaffold, which is crucial for maintaining the barrier function of the transplant. The immunofluorescence staining results demonstrate the ability of the HAAM scaffold to facilitate the formation of cell–cell connections between endothelial cells, as well as cell-scaffold connections between endothelial cells and the carrier scaffold.

### 3.3 Ultrastructure of HCEC-HAAMs

Under SEM, the HAAM scaffold exhibits its unique microscopic morphology: a dense network of interwoven fibers is clearly visible, with these fibers tightly arranged to form a stable mesh-like support system (Figures 4C,D). This structure not only imparts the acellular amniotic membrane with excellent mechanical properties but also provides abundant binding sites for cell attachment on its surface. The collagen fibers and other extracellular matrix components preserved during the decellularization process collectively constitute a microenvironment conducive to cell growth and differentiation. From the observations of HCECs grown on the acellular amniotic membrane scaffold using an electron microscope, the cells exhibited a polygonal morphology, with hexagons being the most typical, which is highly consistent with the natural morphology of corneal endothelial cells *in vivo*. The cell surface was adorned with microvilli. Furthermore, tight cell–cell junctions were formed between adjacent HCECs, which were crucial for maintaining the integrity and function of the cell layer (Figures 4E,H–J).

### 3.4 Gene expression characteristics of HCEC-HAAMs

**Figure 7:**
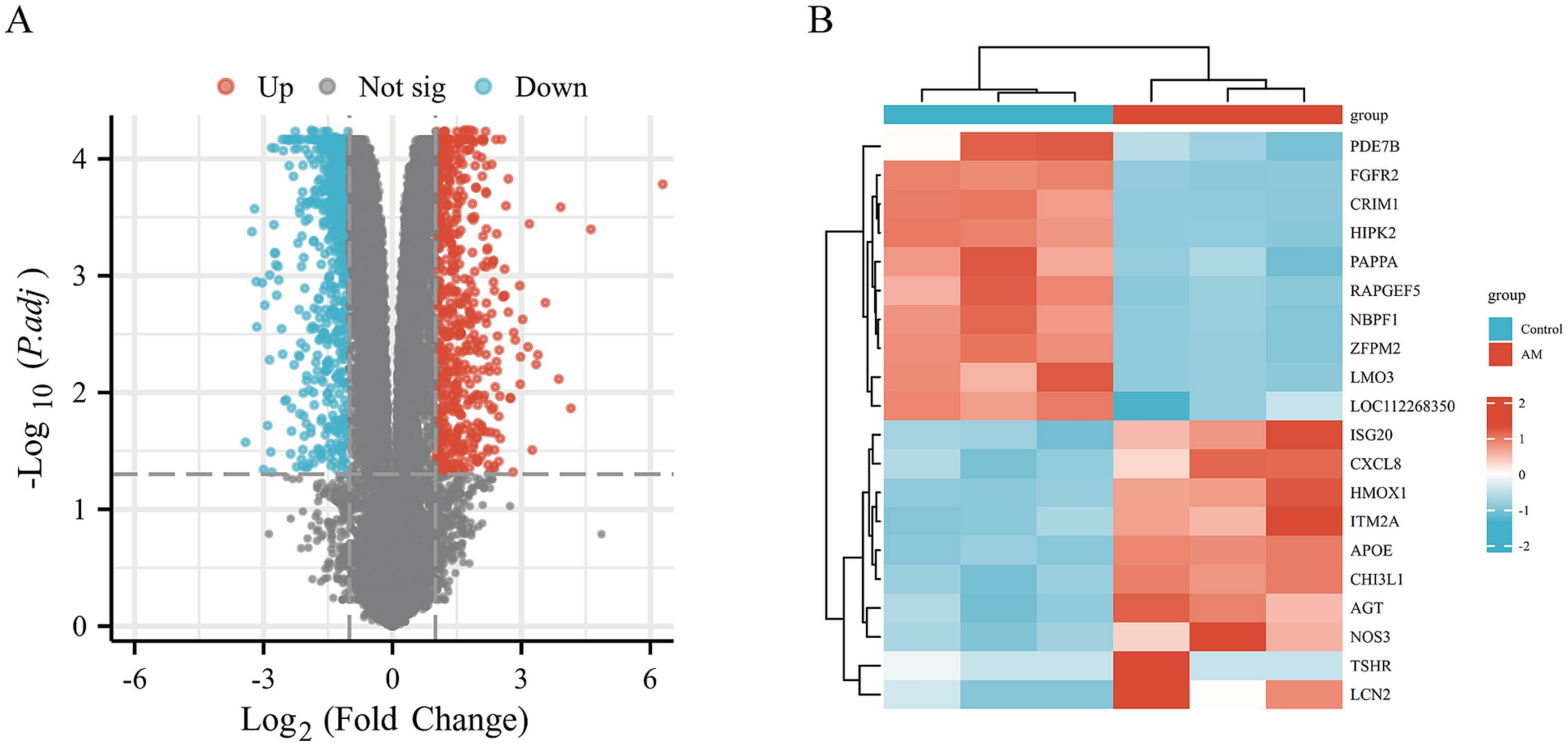
Volcano map and heat map. Differential gene screening **(A)** volcano plot was drawn with the screening threshold set at |logFC| >1 and *p*adj <0.05. **(B)** Simple numerical heatmap of some genes.

To delve into the dynamics of gene expression in HCECs on HAAM scaffolds and the underlying regulatory mechanisms, we used transcriptome sequencing technology coupled with bioinformatics analysis methods. Through high-throughput sequencing, we obtained a comprehensive gene expression profile of HCECs on HAAM scaffolds compared to a blank control group. Differential gene expression analysis revealed significant changes in gene expression between the two groups, with a screening criterion of an absolute logFC value of >1 and an adjusted *p*-value of <0.05. A total of 1,462 genes exhibited more than two-fold changes in expression, among which 634 genes were significantly upregulated and 828 genes were significantly downregulated (Figures 7A,B). These changes not only reflect the positive response of HCECs to the HAAM scaffolds but also indicate the profound influence of the scaffold material on the biological characteristics of the cells.

**Figure 8:**
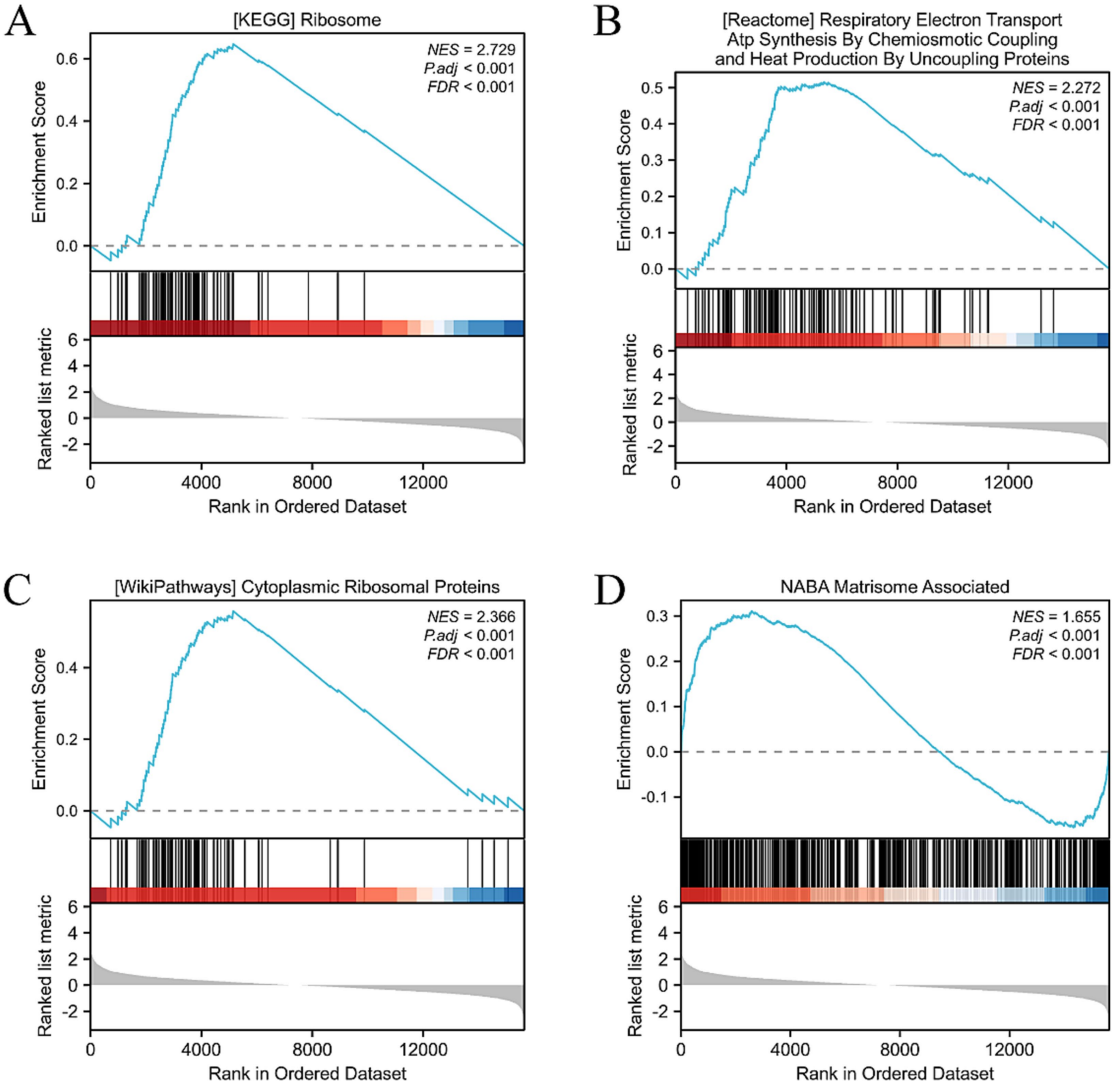
GSEA, GO, and KEGG analysis. **(A)** Activation of the KEGG RIBOSOME pathway has multiple effects on cells, including positive promoting effects such as enhancing protein synthesis efficiency, boosting cell metabolism, and accelerating cell growth and division. **(B)** The NABA MATRISOME ASSOCIATED pathway, which is related to the extracellular matrix (ECM), plays a crucial role in the composition, structure, and function of the ECM. For instance, it involves the synthesis and secretion of matrix proteins and the assembly of matrix proteins and ECM formation. **(C)** Activation of the REACTOME RESPIRATORY ELECTRON TRANSPORT ATP SYNTHESIS pathway may have the following effects: enhanced mitochondrial function: this pathway is an important component of the mitochondrial respiratory chain. After activation, it may improve mitochondrial function, including electron transfer efficiency and ATP synthesis capacity. This will help cells obtain more energy to maintain their life activities. **(D)** Activation of the WP CYTOPLASMIC RIBOSOMAL PROTEINS pathway has multiple effects on cells, mainly involving enhanced ribosome function, adjustment of cell metabolism, and promotion of cell growth and proliferation.

**Figure 9:**
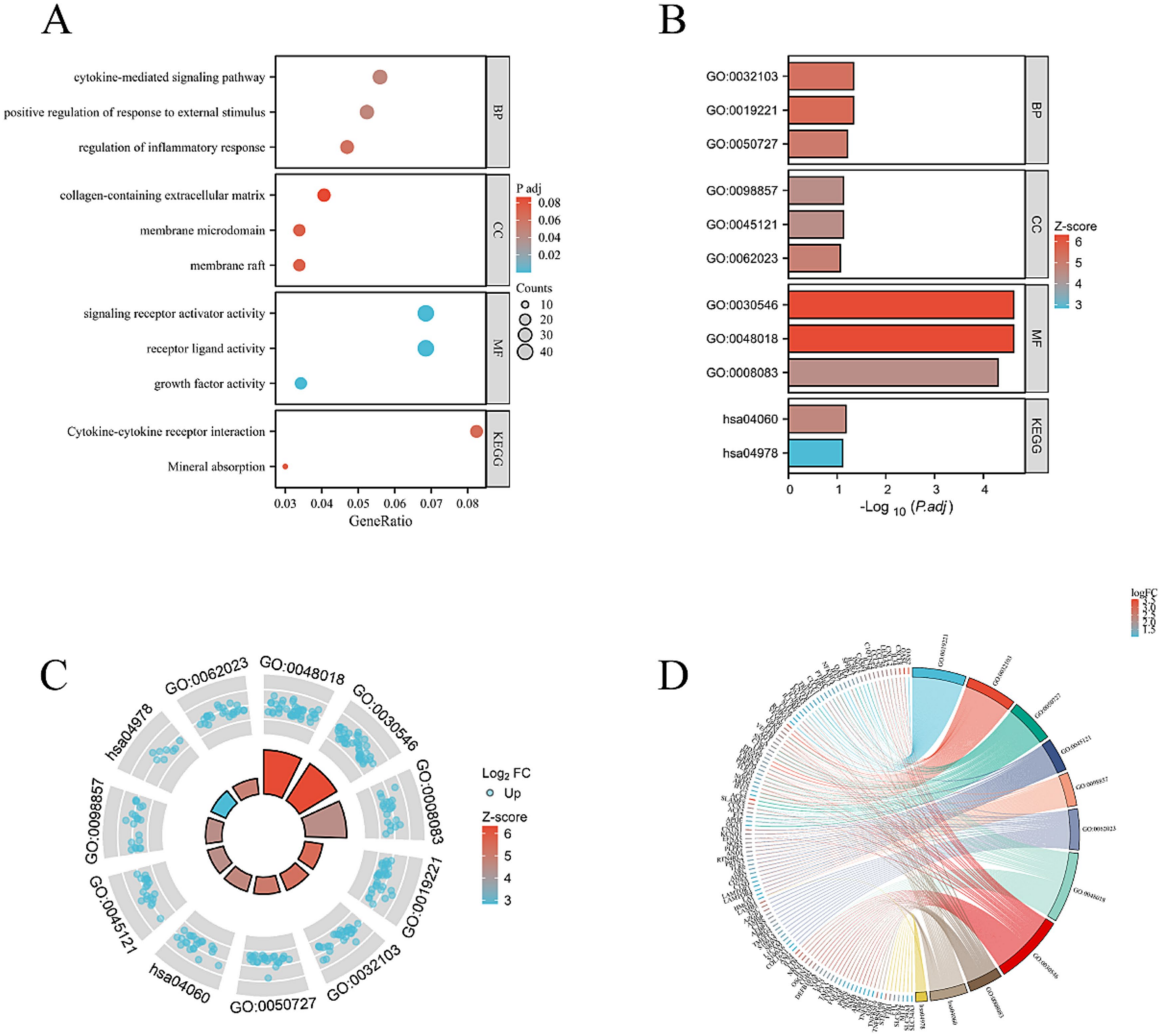
**(A)** BP, CC, and MF. Mainly concentrated on cell metabolism, proliferation, and extracellular matrix synthesis. **(B–D)** Combined analysis of the interested BP, CC, MF, KEGG genes, and LogFC. Bar charts, circle plots, and chord plots are presented.

Using further the gene set enrichment analysis (GSEA) and Kyoto Encyclopedia of Genes and Genomes (KEGG) pathway analysis, we revealed that multiple pathways closely related to cellular metabolism, proliferation, and extracellular matrix (ECM) synthesis were significantly activated. Specifically, the KEGG_RIBOSOME pathway promotes efficient protein synthesis, thereby enhancing the cell’s metabolism, growth, and division. This provides strong support for the rapid proliferation of HCECs on the acellular amniotic membrane scaffold. The NABA_MATRISOME_ASSOCIATED pathway focuses on the composition, structure, and function of the ECM. Its activation facilitates the synthesis, secretion, and assembly of matrix proteins, contributing to the construction of a stable extracellular environment and strengthening interactions between HCECs and the scaffold. The REACTOME_RESPIRATORY_ELECTRON_TRANSPORT_ATP_SYNTHESIS_BY_CHEMIOSMOTIC_COUPLING_AND_HEAT_PRODUCTION_BY_UNCOUPLING_PROTEINS pathway optimizes mitochondrial energy metabolism, enhances ATP synthesis efficiency, and provides sufficient energy support for the cells, ensuring the active state of HCECs on the scaffold. The WP_CYTOPLASMIC_RIBOSOMAL_PROTEINS pathway promotes the enhancement of ribosomal function, accelerates the process of intracellular protein synthesis, and exerts comprehensive positive effects on cellular metabolism, growth, and proliferation (Figures 8A–D). Based on biological process (BP), cellular component (CC), and molecular function (MF), we conducted an in-depth GO and KEGG enrichment analysis, which primarily highlighted enhancements in cellular metabolism, proliferation, and ECM synthesis (Figures 9A–D). These findings not only validate the optimizing effect of the HAAM scaffold on the growth environment of HCECs but also reveal the crucial role of the scaffold material in regulating cellular biological behaviors.

**Figure 10:**
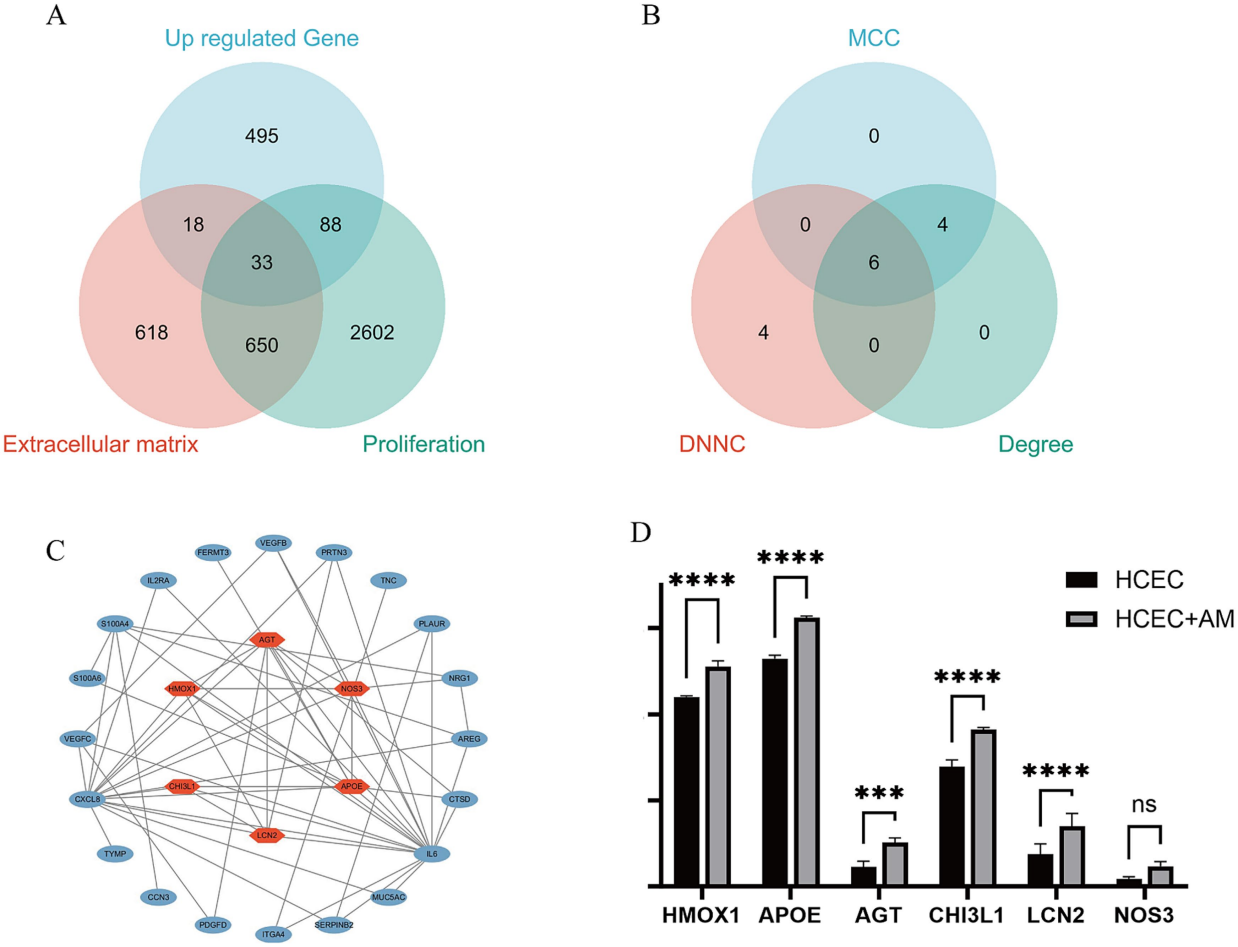
Hub gene screening. **(A)** Based on the previous analysis, the upregulated genes were primarily enriched in biological processes, promoting cell proliferation and extracellular matrix synthesis, and the related pathways were also activated. Therefore, the upregulated genes and the gene sets related to cell proliferation and extracellular matrix in the Genecard database were intersected. Genes that were all protein-coding and had a correlation coefficient greater than 2 were selected, resulting in 33 genes. **(B)** The 33 genes were visualized in Cytoscape, and the top 10 Hub genes were screened using three algorithms: MCC, DNNC, and degree. The intersection of these genes was taken, resulting in six intersection genes. **(C,D)** Visualization expression level of phenotype-related genes and Hub genes.

To identify hub genes central to HCEC proliferation and ECM synthesis, we screened 33 protein-coding genes closely associated with these processes using the GeneCard database (Figure 10A). Subsequently, utilizing Cytoscape software and three algorithms (MCC, DNNC, and Degree) (Figure 10B), we further selected six hub genes from these candidates: AGT, APOE, LCN2, CHI3L1, HMOX1, and NOS3 (Figure 10C). The upregulated expression of these genes is of paramount importance for promoting HCEC proliferation and ECM synthesis on the HAAM scaffold (Figure 10D).

### 3.5 Quantitative real-time PCR results

**Figure 11:**
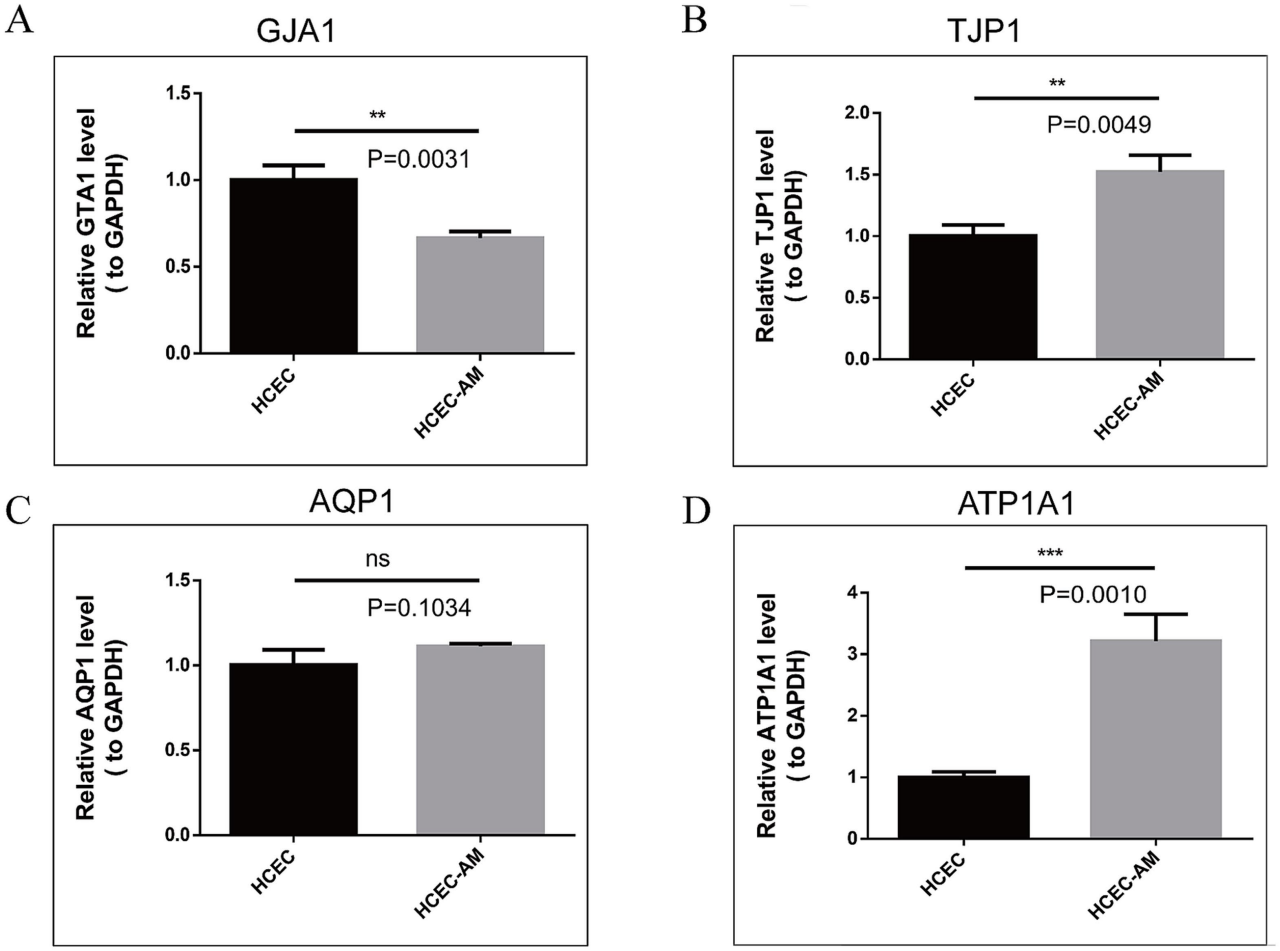
**(A–D)** RT-qPCR results of TJP1, GJA1, AQP1, and ATP1A1. The expression of TJP1 and ATP1A1 genes was high in the amniotic group, the expression of the GJA1 gene was low in the amniotic group, and the expression of AQP1 was not different between the two groups.

The real-time quantitative PCR results (Figures 11A–D) demonstrate that the mRNA expression levels of *TJP1* and *ATP1A1* genes in HCECs cultured on the HAAM scaffold are significantly higher than those in HCECs cultured in Petri dishes (TJP1: 1.50 ± 0.14-fold increase, *p* < 0.01; ATP1A1: 3.22 ± 0.43-fold increase, *p* < 0.01). These findings align with the transcriptome sequencing data and confirm that HAAM enhances the expression of genes critical for barrier integrity (TJP1) and ion transport (ATP1A1). In contrast, GJA1 expression in the HAAM group was lower compared to the control (*p* < 0.01), likely due to the scaffold’s dense collagen structure physically limiting gap junction formation. However, the retained expression level still supports sufficient intercellular communication for coordinated endothelial function. No significant difference was observed in AQP1 expression (*p* = 0.10), indicating that HAAM preserves water channel activity necessary for corneal transparency. Collectively, these results highlight HAAM’s ability to selectively modulate key functional genes while maintaining essential physiological properties of corneal endothelial cells.

## 4 Discussion

In recent years, the thriving development of corneal tissue engineering has opened new avenues in the field of artificial corneas. Carrier scaffold materials are not only a pivotal component of tissue engineering research but also a key determinant of success in tissue construction. The amniotic membrane, derived from placental tissue, possesses a certain degree of toughness while remaining thin and transparent. However, the strong antigenicity of the chorion can lead to graft rejection and dissolution in some cases, as reported by Rotth (47). It is important to note that certain processed chorion-derived products have been successfully used clinically after undergoing specific treatments to reduce antigenicity (48). In our study, the observed graft failure could be attributed to the untreated antigenic components of the chorion, highlighting the importance of adequate decellularization or antigenic removal processes. The HAAM not only retains bioactive components of the amniotic membrane, such as growth factors and cytokines, but also reduces immunogenicity through decellularization, thereby enhancing its safety and biocompatibility (49). Multiple methods are available for decellularizing the amniotic membrane. In addition to the methods described in our study, several methods for removing the cellular components on the amniotic membrane surface have also been reported in the literature. For instance, freeze–thaw methods, mechanical scratching, and enzymatic digestion all aim to destroy and remove cells through physical force or the action of enzymes (36, 49). Each of these methods has its advantages and limitations. Mechanical scraping, for instance, is a relatively simple and inexpensive method but may not achieve complete decellularization and can damage the extracellular matrix. Enzymatic digestion can be more effective in removing cellular components; however, it may also degrade important extracellular matrix proteins if not carefully controlled. In contrast to these methods, our process combines physical agitation with a sequence of chemical treatments to achieve thorough decellularization while minimizing damage to the extracellular matrix. Our process is designed to be relatively straightforward, reproducible, and cost-effective. It utilizes standard laboratory reagents and equipment, making it accessible to a wide range of research and clinical settings. Furthermore, the process can be easily scaled up for large-scale production, which is essential for meeting the growing demand for acellular biomaterials in tissue engineering and regenerative medicine. Innovatively, we utilized HAAM as a scaffold material for corneal endothelial cells and systematically evaluated its biocompatibility, cell adhesion, and proliferation capabilities, as well as the expression of functional genes through a series of *in vitro* experiments.

In terms of selecting seed cells, previous studies have utilized cat and rabbit corneal endothelial cells (50) seeded onto amniotic membrane materials. However, notable biological differences exist between animal and human corneal endothelial cells. To address these differences, we selected the immortalized human corneal endothelial cell line HCEC-B4G12 as our experimental model, which more accurately replicates the biological behavior and functional characteristics of primary human corneal endothelial cells. The HCEC-B4G12 cell line demonstrates a stable genetic background and biological profile, enabling it to maintain consistent proliferation and differentiation capabilities during extended *in vitro* cultivation (51, 52).

First, based on the experimental results, HAAM exhibits good transparency and mechanical properties, which are essential requirements for corneal transplant materials. Through electron microscopy observation and HE staining, as well as Masson staining, we confirmed that, after decellularization, the cellular components on the surface of HAAM were effectively removed, while the internal collagen fiber structure was preserved, providing an ideal matrix environment for the attachment and growth of corneal endothelial cells. Furthermore, the immunogenicity of HAAM was significantly reduced, enhancing its biosafety and potential for clinical application. In this study, we pre-treated the amniotic membrane material with FNC. Fibronectin (FN), a key extracellular matrix glycoprotein, promotes cytoskeletal reorganization and stabilizes focal adhesions by engaging integrins (53), enabling HCECs to firmly attach to the HAAM scaffold. Chondroitin sulfate (CS), a sulfated glycosaminoglycan, complements FN by enhancing ECM structural integrity and providing binding sites for growth factors such as bFGF, which was included in the culture medium. The synergistic effects of FN and CS in FNC thus explain the observed higher cell viability and EdU-positive rates in the HAAM + FNC + HCEC group compared to the control group (Figure 5). In terms of cell compatibility, observations made using an inverted phase contrast microscope and HE staining of frozen sections revealed that HCECs can stably adhere, spread, and form a continuous monolayer on human amniotic membrane allograft (HAAM). This finding suggests that HAAM provides a favorable surface for HCEC attachment. Compared to the current method of cell suspension injection, HAAM allows for controlled cell placement, thereby avoiding the risks of increased intraocular pressure or trabecular meshwork obstruction caused by suspended cells in the anterior chamber. Through CCK-8 and EdU assays, we demonstrated that the proliferative activity of HCECs on HAAM is significantly higher than that of the control group, further confirming the promoting effect of HAAM on cell viability and proliferation. Regarding functional gene expression, immunofluorescence staining results showed that HCECs cultured on HAAM maintained positive expression of the tight junction protein ZO-1, which is essential for maintaining the barrier function and intercellular communication of the corneal endothelial layer. Simultaneously, real-time quantitative PCR results indicated that the expression levels of key genes related to endothelial cell pump function (54), namely aquaporin AQP-1 and the sodium-potassium pump Na^+^/K^+^ ATPase gene (ATP1A1), were upregulated on HAAM. This upregulation contributes to maintaining corneal transparency and water balance. The reduced expression of gap junction protein GJA1 compared to the control group may be associated with the physical barrier formed by the dense fibrous structure of the amniotic membrane (55).

Furthermore, this study utilized transcriptome sequencing and bioinformatics analysis to reveal that the HAAM scaffold regulates the biological behavior of HCECs through multidimensional mechanisms. Differential gene enrichment analysis showed that HAAM significantly activates the ribosome protein synthesis pathway (KEGG_RIBOSOME) and the mitochondrial energy metabolism pathway (REACTOME_RESPIRATORY_ELECTRON_TRANSPORT), thereby enhancing cellular proliferative capacity. Meanwhile, the activation of the NABA_MATRISOME_ASSOCIATED pathway suggests that HAAM promotes cell–matrix interactions by upregulating ECM-related genes (such as COL1A1 and FN1), forming a stable three-dimensional microenvironment (56). Notably, the identified hub genes (such as AGT, APOE, and HMOX1) are not only associated with cell proliferation but may also protect HCECs from damage in the post-transplantation microenvironment through anti-oxidative stress and anti-inflammatory mechanisms, providing targets for subsequent functional optimization of the transplanted grafts (57–59).

Considering current advancements in the field, our research has made significant progress in the application of HAAM as a scaffold material for corneal endothelial cells. Compared to traditional corneal transplantation surgery, corneal endothelial grafts constructed using HAAM offer several advantages such as a wide range of donor sources, low immunogenicity, and good biocompatibility. Furthermore, HAAM can serve as an extracellular matrix material, providing an ideal microenvironment for the attachment and growth of corneal endothelial cells. This helps to address issues such as low cell survival rates and inadequate matrix synthesis capacity observed in traditional single-cell transplantation approaches.

However, this study has several limitations. For example, the cell line we used in the current study is immortalized human corneal endothelial cells, which may exhibit some differences in biological characteristics compared to primary corneal endothelial cells. Future studies should further validate the effects of HAAM on primary corneal endothelial cells. Additionally, although we have obtained positive results in *in vitro* experiments, the long-term efficacy and safety of HAAM in clinical applications still require further evaluation.

## 5 Conclusion

In summary, our research provides new ideas and methods for advancing corneal endothelial cell replacement therapy. In the future, we will continue to optimize and improve the graft, further exploring its clinical potential and value to provide safer and more effective treatment options for patients with corneal endothelial diseases.

## Data Availability

Raw data have been deposited to National Center for Biotechnology Information (NCBI) under the BioProject number PRJNA1271274.
