# Supplementary material for: Research on the construction of corneal endothelium transplantation with acellular amniotic membrane as a scaffold
Source: Front Med (Lausanne). 2025 Jul 1;12:1592123. doi: 10.3389/fmed.2025.1592123 (PMC12259565; doi:10.3389/fmed.2025.1592123)
Supplement: Supplementary file 1 [file Table_1.docx]

Supplement Table 1. Primer used for Reverse transcription PCR

| **Gene** | **Forward primer (5’-3’)** | **Reverse primer (3’-5’)** | **Accession no.** | Tm(℃) |
| --- | --- | --- | --- | --- |
| GJA1 | TCTTCATGCTGGTGGTGTCC | ACCACTGGTCGCATGGTAAG | NM_000165.5 | 60 |
| TJP1 | TAACAGAAGGAGTGAGAAGATTTG | TGTGACTTTAGTAGGTTTAGCAGG | NM_001301025.3 | 60 |
| AQP1 | CTACGACTTCATCCTGGCCC | AGGAAGCTCCTGGAGTTGATG | NM_001329872.2 | 60 |
| ATP1A1 | TTCAGCTACCTGGCTTGCTC | GACTCAGAGGCATCTCCTGC | NM_000701.8 | 60 |
| GAPDH | CGCTGAGTACGTCGTGGAGTC | GCTGATGATCTTGAGGCTGTTGTC | NM_001357943.2 | 60 |
